# Advanced Gastric Cancer With Cutaneous Metastases, With Effective Relief From Immunotherapy: Case Report

**DOI:** 10.1155/cris/7879668

**Published:** 2026-10-30

**Authors:** Hongtao Wang, Pengfei Yu, Xiangliu Chen, Chengkang Zhu, Guangyu Ding, Shengxin Fang, Zequan Wu, Han Chen, Tengjiao Chai, Ling Huang

**Affiliations:** ^1^ Zhejiang Chinese Medical University Hangzhou 310053 Zhejiang China zcmu.edu.cn; ^2^ Department of Gastric Surgery Zhejiang Cancer Hospital Hangzhou Institute of Medicine (HIM) Chinese Academy of Sciences Hangzhou 310022 Zhejiang China cas.cn; ^3^ Postgraduate Training Base Alliance of Wenzhou Medical University (Zhejiang Cancer Hospital) Hangzhou 310022 Zhejiang China zchospital.com

**Keywords:** cutaneous metastasis, gastric cancer, immunochemotherapy, signet-ring cell carcinoma

## Abstract

Cutaneous metastases from gastric cancer are rare (<1%) and typically indicate advanced disease with poor prognosis. We report a unique case of a 53‐year‐old male presenting with an asymptomatic left temporal erythematous nodule, later diagnosed as metastatic gastric adenocarcinoma with signet‐ring cell features. Initial skin biopsy revealed adenocarcinoma (CDX2+/CK20+/CK7+), prompting gastrointestinal evaluation, which confirmed poorly differentiated gastric adenocarcinoma (30% signet‐ring cell component). The patient received neoadjuvant immunochemotherapy (paclitaxel+S‐1+sintilimab), followed by laparoscopic radical gastrectomy (D2 lymphadenectomy) and resection of the skin lesion. Postoperative pathology showed Becker grade 2 regression in the primary gastric tumor and Mandard grade 3 regression in the cutaneous metastasis. The surgical specimen showed metastasis in 20 of 65 lymph nodes and was staged ypT4aN3bM1 (AJCC 8th edition). Biomarker assessment demonstrated high Claudin18.2 expression (~90%) but low PD‐L1 expression (CPS ~1). Postoperative surveillance showed no radiographic local recurrence or distant metastasis through April 2025; however, leptomeningeal metastasis was confirmed by cerebrospinal fluid (CSF) cytology in May 2025. This case illustrates substantial early tumor regression with perioperative chemoimmunotherapy in an unusual metastatic presentation while also demonstrating that early response does not necessarily translate into durable disease control.

## 1. Introduction

Gastric cancer is the fifth most common malignancy and the fifth leading cause of cancer‐related deaths worldwide [1]. While the majority of patients present with locoregional or distant metastases to the liver, peritoneum, or lymph nodes, cutaneous metastases are exceedingly rare, occurring in less than 1% of cases [2]. When present, they most commonly manifest as umbilical nodules (Sister Mary Joseph nodules) or abdominal wall lesions, whereas metastases to the scalp or face are exceptionally uncommon [3, 4].

Cutaneous metastases from gastric adenocarcinoma typically indicate advanced disease and poor prognosis, with a median survival of 3–6 months after diagnosis [5]. The most frequent histological subtype associated with skin dissemination is signet‐ring cell carcinoma, which exhibits aggressive biological behavior and a high propensity for diffuse peritoneal and distant metastasis [6]. Due to their nonspecific clinical presentation, these lesions are often initially misdiagnosed as benign dermatological conditions, leading to delayed diagnosis and treatment [7].

Recent advances in immunotherapy and targeted therapies have provided new treatment options for advanced gastric cancer, particularly in PD‐L1‐positive or microsatellite instability‐high (MSI‐H) tumors [8]. Additionally, HER2‐targeted therapy remains the most established targeted approach in clinical practice. For HER2‐positive gastric cancer (IHC 3+ or FISH+), trastuzumab combined with chemotherapy significantly improves survival (median OS: 13.8 vs. 11.1 months) [9] and is now the standard first‐line regimen globally.

Although Claudin18.2‐targeted agents (e.g., zolbetuximab) show promise in clinical trials [10], they are not yet approved for routine clinical use in China. In contrast, HER2‐targeted therapy has established a mature clinical framework in gastric cancer [9]. However, the role of these novel treatments in gastric cancer with cutaneous metastases remains poorly understood due to the rarity of such cases.

Here, we present a highly unusual case of gastric signet‐ring cell carcinoma initially manifesting as a left temporal cutaneous metastasis, which demonstrated a significant response to combined SP (paclitaxel+S‐1) chemotherapy and anti‐PD‐L1 immunotherapy. This case highlights the diagnostic challenges, therapeutic implications, and prognostic considerations associated with this rare metastatic pattern.

## 2. Case Presentation

The patient is a 53‐year‐old male who inadvertently noticed a red patch on the skin of his left temporal region in July 2024. He sought treatment at Jiangxi Provincial Dermatology Hospital, where a pathological biopsy of the lesion suggested adenocarcinoma of gastrointestinal origin. On July 20, 2024, he was referred to our hospital for further treatment.

At the initial visit, an erythematous nodule with localized changes was observed on the left temporal skin. The skin temperature was not elevated, and no obvious ulceration, erosion, or exudate was present. On palpation, the mass was fluctuant and exhibited tenderness and pruritus (Figure 1A). Subsequent a whole‐body PET‐CT scan showed irregular thickening of the skin in the left temporal region with mild FDG metabolic activity (SUVmax = 3.3), as well as slight thickening of the gastric wall on the greater curvature of the gastric body with mild FDG metabolic activity (SUVmax = 2.6) (Figure 2A). A CT scan confirmed mild thickening of the gastric wall at the greater curvature of the gastric body (Figure 3A). No additional visceral or distant metastatic lesions were documented on the baseline whole‐body PET‐CT. Because the cutaneous lesion represented pathologically confirmed metastatic disease, the patient had metastatic gastric cancer at presentation. In July 2024, a pathological biopsy of the left temporal skin lesion performed at Jiangxi Provincial Dermatology Hospital indicated adenocarcinoma, likely of gastrointestinal origin. Immunohistochemistry (IHC) showed tumor cells positive for CDX2(+), CEA(+), CK20(+), CK7(+), CAM5.2(+), and Ki‐67 (+, ~20%) while negative for TTF‐1(−), P63(−), CK5/6(−), and CD31(−). Combined with H&E morphology, metastatic gastrointestinal adenocarcinoma was diagnosed (Figure 4A–F). Based on the initial imaging and pathological findings, the patient was clinically staged as cTXNXM1 before neoadjuvant treatment, reflecting the presence of a cutaneous metastatic lesion without sufficient information to determine the primary tumor (T) and regional lymph node (N) categories. Gastroscopy on July 20, 2024, at our hospital confirmed gastric body carcinoma. Pathology revealed poorly differentiated adenocarcinoma of the gastric body with a signet ring cell carcinoma component. Immunohistochemical staining showed retained expression of all mismatch repair proteins (hMLH1, hMSH2, hMSH6, PMS2), HER2 negativity (Score 0), a PD‐L1 (22C3) Combined Positive Score (CPS) of less than 1, negative EBER in situ hybridization, and moderate to strong Claudin 18.2 expression in ~90% of tumor cells (Figure 5A–H). These findings confirmed secondary cutaneous metastasis from gastric carcinoma. Colonoscopy was not performed. No additional gastric lesions were identified on gastroscopy.

**Figure 1:**
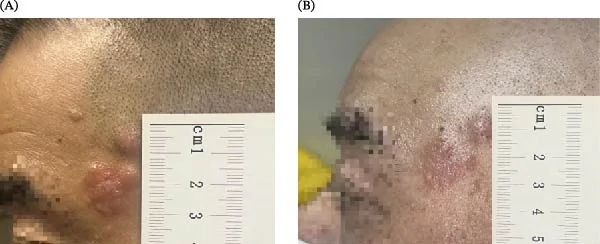
(A) Image of skin lesions on July 2024. (B) Image of skin lesions on September 2024.

**Figure 2:**
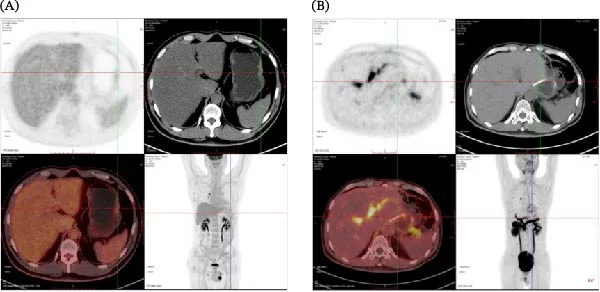
(A) PET‐CT images on July 2024. (B) PET‐CT images on April 2025.

**Figure 3:**
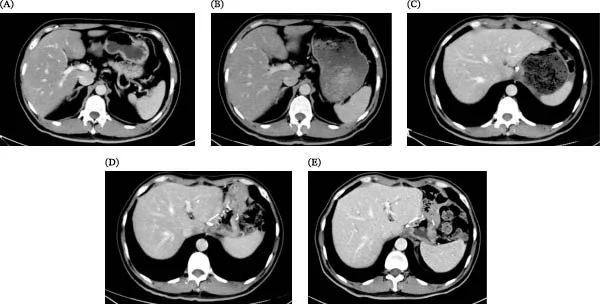
CT variation diagram. (A) CT images on July 24, 2024. (B) CT images on October 7, 2024. (C) CT images on November 26, 2024. (D) CT images on March 3, 2025. (E) CT images on April 3, 2025.

**Figure 4:**
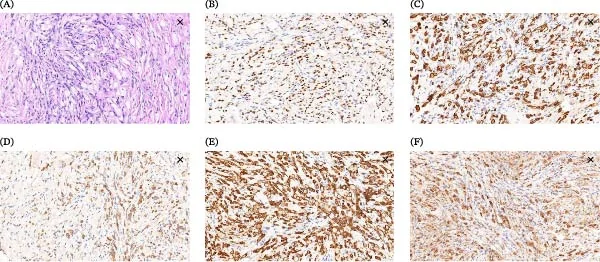
(A) Pathological images of the skin lesions (H&E staining). (B) Immunohistochemical CDX‐2 pathological images. (C) Immunohistochemical CK‐20 pathological images. (D) Immunohistochemical CK‐7 pathological images. (E) Immunohistochemical CAM5.2 pathological images. (F) Immunohistochemical CEA pathological images.

**Figure 5:**
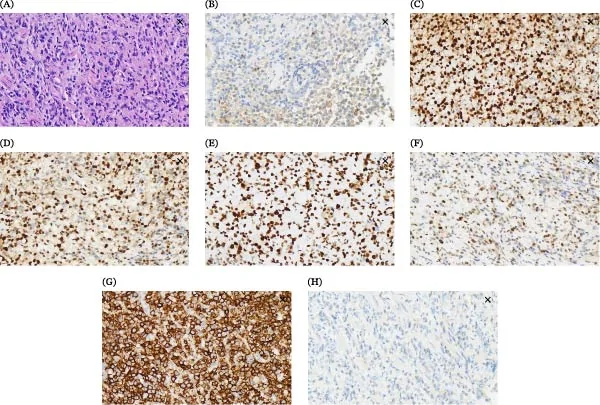
(A) Pathological images of the primary lesion of gastric cancer (H&E staining). (B) Immunohistochemical PD‐L1 pathological images. (C) Immunohistochemical MLH1 pathological images. (D) Immunohistochemical MSH2 pathological images. (E) Immunohistochemical MSH6 pathological images. (F) Immunohistochemical PMS2 pathological images. (G) Immunohistochemical Claudin18.2 pathological images. (H) Immunohistochemical Her‐2 pathological images.

From August 2 to September 12, 2024, the patient received three cycles of SP chemotherapy combined with anti‐PD‐L1 therapy (Paclitaxel 240 mg, Tegafur‐Gimeracil‐Oteracil 60 mg, and Sintilimab 200 mg). The three cycles were administered every 3 weeks (q3w) (Figure 6). No clinically significant immune‐related adverse events occurred during treatment. Treatment response assessment indicated partial remission (Figure 3B), with significant improvement in the left temporal skin lesion (Figure 1B). Following this, on October 22, 2024, the patient underwent laparoscopic‐assisted radical gastrectomy (distal subtotal gastrectomy with D2 lymphadenectomy + Billroth II reconstruction + lysis of intestinal adhesions) and resection of the skin lesion at our institution. Postoperative pathology of the gastric specimen confirmed adenocarcinoma with poorly cohesive carcinoma and signet ring cell carcinoma components (Borrman type: infiltrating ulcerative type). Metastasis was present in 20 out of 65 lymph nodes. Tumor treatment response (NCCN criteria): Grade 2 (minor regression). Pathological staging (AJCC 8th edition): ypT4aN3bM1. Pathology of the resected left temporal skin lesion showed poorly differentiated carcinoma with degeneration in the dermis, surrounded by fibrotic stroma with degeneration, collagenization, and dense lymphocytic/inflammatory infiltrate, including foamy histiocytes (consistent with metastatic poorly differentiated adenocarcinoma, based on history and morphology). IHC results: E‐cad(+), Ki‐67 (+, ~60%), MMR proteins (hMLH1[+], hMSH2[+], hMSH6[partial+], PMS2[+]), Her2(0), EBER(−), PD‐L1 (E1L3N) ‐ Negative (CPS: ~1), and Claudin18.2 (moderate‐strong+, ~90%).

**Figure 6:**
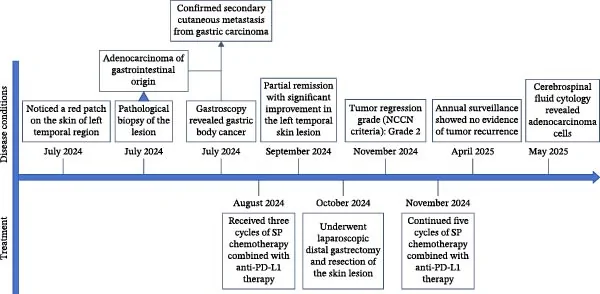
Treatment process diagram.

After surgery, the patient received five cycles of adjuvant SP chemotherapy combined with anti‐PD‐1 therapy from November 27, 2024, to March 3, 2025. The chemotherapy and immunotherapy regimens were consistent with those administered before surgery, with one cycle being administered every 3 weeks. No obvious immune‐related adverse events, dose modifications, or treatment delays were observed during the adjuvant treatment. Regular imaging surveillance (CT and PET‐CT) from November 2024 to April 2025 showed no evidence of local recurrence or distant metastasis (Figures 2B and 3C–E). Routine blood tests, liver function, and serum tumor markers remained within normal ranges (Figure 7A–C). The patient achieved ~6 months of postoperative disease control from November 2024 to May 2025. In May 2025, the patient developed bilateral lower‐limb weakness. Cerebrospinal fluid (CSF) cytology identified adenocarcinoma cells, confirming leptomeningeal metastasis. The patient continues regular follow‐up.

**Figure 7:**
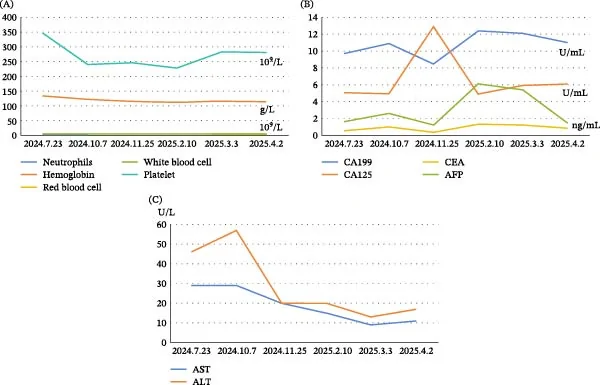
(A) Blood routine change map, (B) tumor markers change map, (C) liver function change map.

## 3. Discussion

Cutaneous metastasis from gastric adenocarcinoma is a rare phenomenon, occurring in only 0.8%–1.3% of advanced gastric cancer cases and typically signifies disseminated disease with a dismal prognosis [1, 7]. In a retrospective study of 72 cases, nodular skin lesions were the most common presentation (45.8%), frequently located on the chest or abdominal wall (56.9%), with multiple lesions in 61.1% of patients. The median survival was only 6 months, although patients who underwent surgical resection of isolated metastases achieved survival up to 48 months [11]. This case illustrates an exceptional presentation of gastric cancer initially manifesting as a solitary left temporal skin lesion, later confirmed as metastatic adenocarcinoma of gastrointestinal origin through IHC (IHC: CDX2+, CK20+, and CK7+). Notably, the patient exhibited a significant response to neoadjuvant chemoimmunotherapy (SP regimen + anti‐PD‐L1), achieving partial regression of both the primary gastric tumor and skin metastasis.

Skin involvement often precedes primary tumor diagnosis in 30%–40% of cases [4]. However, delayed cutaneous metastasis has also been reported, with intervals as long as 15 years after curative gastrectomy, as in a case of left inguinal skin metastasis that appeared 15 years after surgery [12]. Our patient’s initial presentation with a temporal nodule highlights the critical role of IHC (e.g., CDX2 for gastrointestinal origin) in guiding the diagnostic workup for occult malignancies [13, 14]. The absence of visceral symptoms underscores skin lesions as potential sentinel signs of internal cancers.

Skin metastasis from gastric cancer often indicates a poor prognosis. Although the prognosis is usually poor, surgical resection of isolated cutaneous lesions may lead to prolonged survival. One case described a 60‐year‐old patient with six cutaneous metastases (scalp and thigh) who underwent resection of all lesions; the patient remained disease‐free for 6 years, and after local recurrence at 7.5 years, underwent additional surgery and achieved durable remission [15].

While nodular lesions are most common, cutaneous metastasis may mimic inflammatory or infectious skin diseases. For example, a recent report described a patient with gastric cancer presenting with erythematous, indurated skin lesions resembling cellulitis, later confirmed as cutaneous metastasis; the patient died only 7 days after the diagnosis [16]. In another report, a 60‐year‐old woman initially presented with pruritic, eczema‐like lesions on the face, which were misdiagnosed as dermatitis. Skin biopsy later revealed metastatic gastric adenocarcinoma. After systemic chemotherapy, the lesions regressed completely, achieving a clinical complete response [17]. An unusual presentation involves alopecia neoplastica, in which metastatic nodules lead to localized scalp alopecia. A case published in the Cleveland Clinic Journal of Medicine reported a patient with CK7+/CK20+/CDX2+ gastric adenocarcinoma who presented with round, smooth scalp nodules with hair loss; the patient declined treatment and died 1 year later [18].

Iatrogenic tumor implantation is a well‐recognized mechanism of cutaneous metastasis following invasive procedures, including surgery, biopsy, drainage, or catheter insertion [19]. A previous case report by Karayiannakis et al. clearly demonstrated that cutaneous metastases may develop directly at surgical or puncture sites as a result of accidental seeding of malignant cells during manipulation [20]. This mechanism highlights the importance of careful surgical technique and protective measures to minimize tumor spillage during gastroscopy, biopsy, or abdominal surgery. In our patient, no prior invasive procedures were performed before the appearance of the skin lesion; thus, iatrogenic implantation was not involved.

Hematogenous spread to skin‐rich regions (scalp and abdomen) is favored over lymphatic dissemination in gastric cancer [2, 19, 21]. The temporal location here is unusual; most reported cases involve abdominal or umbilical skin (so‐called “Sister Mary Joseph nodule”) [22–24]. The late emergence of leptomeningeal metastasis (detected via CSF cytology) reflects the disease’s propensity for serosal surfaces and neurotropic spread in signet ring carcinomas [25].

The marked regression of the skin lesion after neoadjuvant therapy (Figure 1B) demonstrates a substantial treatment response in the cutaneous metastasis. However, because the patient received paclitaxel/S‐1 together with sintilimab, the relative contribution of chemotherapy and immunotherapy cannot be determined from this single case, and the findings should not be interpreted as evidence of synergistic efficacy. The tumor was mismatch repair proficient and had low PD‐L1 expression (CPS ~1) [26]. The high Claudin18.2 expression (90%, strong+) further offers a therapeutic avenue for investigational agents like zolbetuximab in future relapses [27], but the present case does not provide evidence for the efficacy of Claudin18.2‐directed therapy in this setting.

Historically, cutaneous metastasis from gastric cancer has been associated with poor outcomes. In this patient, no radiologically evident local recurrence or distant metastasis was observed for ~6 months after surgery. However, leptomeningeal metastasis was subsequently confirmed by CSF cytology in May 2025, indicating that the initial disease control was not durable. Rather, it illustrates substantial early tumor regression, followed by clinically significant progression.

Overall, cutaneous metastasis from gastric adenocarcinoma signifies an advanced systemic disease and generally carries a poor prognosis. In this patient, combined systemic therapy and surgery were associated with marked regression of the cutaneous lesion and partial regression of the primary tumor. The case also emphasizes the value of early skin biopsy and IHC when an atypical cutaneous lesion precedes the recognition of an internal malignancy. However, the subsequent leptomeningeal metastasis highlights the aggressive biology of this disease and the limitations of using the early response as a surrogate for durable control.

This single case provides a hypothesis‐generating observation rather than evidence for a change in the management paradigm. Although perioperative chemoimmunotherapy achieved substantial pathological and clinical regression in both the primary tumor and the cutaneous metastasis, the patient later developed leptomeningeal disease. Larger case series, retrospective cohorts, and prospective studies are needed to determine whether immunotherapy‐containing multimodal strategies provide reproducible benefit in gastric cancer with cutaneous metastases and to identify patients most likely to benefit.

## Author Contributions

Pengfei Yu conceived the case report. The first draft of the manuscript was written by Hongtao Wang. Material preparation, data collection, and analysis were performed by Hongtao Wang, Chengkang Zhu, Guangyu Ding, Ling Huang, Shengxin Fang, Zequan Wu, Han Chen, and Tengjiao Chai. Xiangliu Chen contributed to the revision of the manuscript.

## Funding

No funding was received for this research.

## Disclosure

All the authors reviewed the manuscript. All authors have read and agreed to the published version of the manuscript. No financial or non‐financial benefits have been received from any party related directly or indirectly to the subject of this manuscript. Pharmaceutical products mentioned (e.g., sintilimab, S‐1) are cited for scientific accuracy without endorsement.

## Ethics Statement

The study was conducted according to the guidelines of the Declaration of Helsinki and approved by the Zhejiang Cancer Hospital. For deceased patients in historical comparisons, waiver of consent was granted by the ethics committee due to the retrospective nature of data collection.

## Consent

Written informed consent was obtained from the patient for the publication of this case report and accompanying images.

## Conflicts of Interest

The authors declare no conflicts of interest.

## Data Availability

All relevant data supporting the results of this case report are available from the corresponding author upon reasonable request.
